# Critical Review of Neurobiological Evidence for Relationships Between Social Isolation, Loneliness and the Risk of Developing of Alzheimer's Disease: A New Model

**DOI:** 10.1155/jare/9924448

**Published:** 2025-07-02

**Authors:** Jacob K. De Puit, Kirsten L. Challinor

**Affiliations:** School of Behavioural and Health Sciences, Australian Catholic University, Strathfield, New South Wales, Australia

**Keywords:** Alzheimer's disease, dementia, depression, loneliness, neurobiology, social isolation

## Abstract

**Background:** It is known that people who are socially isolated and lonely are more likely to develop Alzheimer's disease (AD) than people who are neither socially isolated nor lonely. This work addresses the direct impact of socially isolation and loneliness on the brain.

**Aim:** To review the neurobiological evidence on the relationships between social isolation, loneliness and AD pathogenesis.

**Method:** Neurobiological literature in relation to social isolation, loneliness and how these factors impact risk of AD was reviewed. A new model providing a framework to describe the links between these pieces of evidence was created.

**Results:** Social isolation contributes to AD pathogenesis via neuroinflammation and stress pathways. Loneliness is linked to AD risk mainly through its strong association with depression.

**Conclusion:** Although social isolation and loneliness are typically linked together, they should be considered separately in the context of AD because, neurobiologically, social isolation is more closely linked to AD than loneliness is linked to AD.

**Implications:** Clinicians should be cognisant that socially isolated people who are not lonely may be at higher risk for AD than people experiencing loneliness who are not socially isolated. Measures of depression are likely more appropriate for appraising AD risk than measures of loneliness.

## 1. Introduction

There are 55 million people living with dementia worldwide, and this number is growing by 10 million each year [1]. Dementia refers to a cognitive decline and is caused by various diseases [1]. The most common type of dementia (60%–70%) is Alzheimer's disease (AD). AD is typically age-related and is characterised by broad symptoms of cognitive decline including memory loss, language difficulties and behaviour change. The precise mechanisms behind AD remain unclear, but it is thought to be caused by neuron cell death resulting from a cascade of immune dysregulation [2] via mechanisms involving brain immune cell dysfunction over time [3].

### 1.1. Risk Factors and AD

As long as the mechanisms behind the development of AD remain unclear, practitioners rely on eliminating risk factors to protect against pathogenesis. Eliminating risk factors for people who are at risk of AD reduces the chance and speed of pathoprogression, and addressing risk factors in people who are early AD stages reduces symptom severity as the condition develops. Addressing risk factors is the best defence against AD that currently exists for most people.

Risk factors are typically identified by observational evidence or identifying pathological processes which occur in AD and preventing those processes before the disease manifests. Social isolation and loneliness are both linked with AD observationally [4, 5]. People living with AD (PLWAD) who are socially isolated experience affective dysregulation [6] and neuropsychiatric symptoms [7] which negatively impact quality of life [8]. Isolated individuals present elevated neuropsychiatric symptoms such as aggression, agitation, aberrant motor activity and apathy [9–11], resulting in poor outcomes for PLWAD and their carers in community, clinical and residential aged care home settings [8, 12].

### 1.2. The Neurobiology of AD

AD begins with the formation of Aβ-amyloid plaques, which are plaque deposits of various proteins including Aβ protein, which normally has a role in reducing synaptic activity [13]. Aβ-amyloid plaques are consumed by resident immune cells in the brain, called microglia [14]. When activated by phagocytosis of Aβ-amyloid proteins, microglia become enlarged and dysfunctional and release inflammatory signals into the brain. For as long as the Aβ-amyloid proteins are in the brain, this response compounds upon itself and affects more microglia across the brain. As this continues, the tau protein, which is another important part of the AD pathogenic process, becomes toxic (via hyperphosphorylation) and begins to cause cell death [15]. Normally, tau is part of the cell skeleton and transport system, but it is removed from the cell structure when it becomes toxic and disrupts normal cell processes and architecture [16]. Eventually, toxic tau builds up in the cell, causes tangles (called neurofibrillary tangles), and leads to cell death. Tau is especially important in cells with long cell bodies, so this process is particularly damaging to neuron cells [16]. Currently, this process is thought to be the main contributing mechanism through which buildup of Aβ amyloid proteins leads to brain degeneration.

### 1.3. Social Isolation and Loneliness

Social isolation and loneliness are often observed in humans at some point during the lifespan. Humans, as highly social animals, attract and are attracted to group formation innately. Our brains have developed to facilitate and reflect our group-forming behaviours to the point that we experience profound repercussions when we are isolated [17].

A person is socially isolated when they lack a sense of belonging socially; that is, they lack real or perceived social interactions either due to a physical lack of people with whom to engage or through a lack of fulfilling and quality relationships [18]. Social isolation can be intentional and restorative, but the term usually refers to a type of solitude which is unwanted and unhealthy [19–21]. The unhealthy symptoms of social isolation include increased stress inflammatory issues, and cognitive decline, and are thought to be due at least in part to the lack of engagement of the specialised human social brain [19, 22].

Loneliness is more difficult to define. The author of [23] defined loneliness as a “lack of affection in existing relationships and poor-quality social relations.” It is possible to capture the forms of loneliness via categorisation according to the cause [24]. Situational loneliness is a product of deficiency of interpersonal interactions in one's environment, whether realised or perceived. Developmental loneliness proceeds from an inability for a person to balance their need for relationships with solitude, such that they lose sufficiently profound meaning from their life to cause affective responses of emptiness and loneliness. Internal loneliness is perception-based only and occurs when a person's self-concept causes them to feel isolated regardless of their social interactions.

In practice, loneliness can be defined as a clinical symptom [25]. However, recent approaches have begun to consider loneliness itself a disease, particularly in the 60+ years demographic [24].

A useful working definition of loneliness, inspired by Hawkley and Cacioppo's [26] definition, is “a potentially pathological, distressing feeling accompanying the salient or not-salient perception that one's social needs are not being met by the quantity or quality of one's social relationships.”

Social isolation and loneliness are conceptually closely linked, since a person can perceive themselves as lonely when they are socially isolated [27]. Both are important considerations for people who are cognitively impaired, particularly in the field of dementia because of the associated negative affective, cognitive and neurobiological outcomes.

### 1.4. Social Isolation and AD

The elevated incidence of social isolation and loneliness in PLWAD during COVID-19 pandemic-related lockdown events has drawn attention to the impacts of social isolation in dementia. Much of the population has been affected by lockdowns, but government-mandated social isolation has disproportionately affected the older population [11, 28, 29]. PLWAD and people at risk of AD experienced negative health outcomes because of social isolation, including steeper rates of memory decline [30], neuropsychiatric symptoms [31] and cognitive function [32]. Accurate appraisal of AD risk from social isolation and loneliness is therefore highly important in the current landscape [33].

With the current impetus on social isolation and loneliness, it is important to fully understand how people may have been affected by COVID-related isolation. Whilst observational evidence is useful, the direct impact on the brain must be understood from a fundamental level using an inclusive model. This paper therefore aims to review the neurobiological evidence on the relationships between social isolation, loneliness and AD pathogenesis and to use the evidence to propose a new model on the contribution of social isolation and loneliness in the pathology of AD.

### 1.5. Review Structure

This review focusses on how social isolation and loneliness contribute to the most common type of dementia, AD. A summary of evidence follows on why social isolation and loneliness may be useful to consider separately in this context. Next, the neurobiological evidence on social isolation and loneliness in AD is reviewed. Depression is explored as an important moderator for the relationship between loneliness and AD in context of the underlying mechanisms. Finally, the proposed model is discussed with recommendations for clinical practice.

### 1.6. The Model

**Figure 1:**
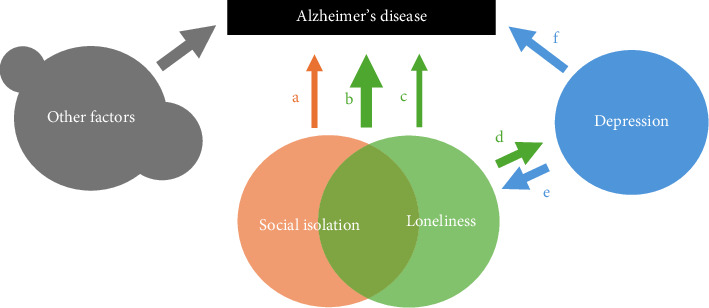
Proposed model of social isolation and loneliness in Alzheimer's disease. Circles represent disorders or conditions as they relate to Alzheimer's disease (AD) and contain the active neurobiological contributors to any associations between factors or to AD. Arrow weight indicates approximate relative strength. Arrows do not imply causation. Overlap between circles represents copresentation with both conditions. Arrow a: impact of social isolation on incidence or severity of AD. Arrow b: impact of co-occurring social isolation and loneliness on incidence or severity of AD. Arrow c: impact of loneliness on incidence or severity of AD. Arrow d: impact of loneliness on incidence or severity of depression. Arrow e: impact of depression on incidence or severity of loneliness. Arrow f: impact of depression on incidence or severity of AD. Depression is included as a possible moderating factor between loneliness and AD. ‘Other factors' represent other risk factors contributing to AD outside the scope of this review.

In Figure 1, we present our neurobiological perspective model. It focuses only on the central factors in social isolation, loneliness and AD. Circles represent contributing disorders or conditions to AD (represented by box at top). Arrows represent the direction of relationships between each factor. Overlapping circles are used to explain the effect of copresentation of both factors. The ‘other factors' component represents contributing factors to AD outside the scope of this review, such as genetic factors, traumatic brain injury, diet or immunological factors [8, 34]. This review will address each element of the model in subsequent sections for clarity.

## 2. Review of Evidence on the Relationship Between Social Isolation, Loneliness and Risk of Developing AD

### 2.1. Is It Appropriate to Consider Social Isolation and Loneliness Interchangeable? (Social Isolation and Loneliness Circles)

The section of the model where social isolation and loneliness meet (Figure 1) represents when both occur together. Social isolation and loneliness are intuitively similar and are expected to co-occur, so are sometimes linked together in research [20, 35, 36]. This approach is frequently adopted in clinical settings, where assessments view these factors interchangeably [37]. However, social isolation and loneliness are often not correlated with each other. For example, Coyle and Dugan [20] found that the correlation between loneliness and social isolation in their large sample (11,825 participants) was only *r* = 0.201. Some study results with large samples (15,000 participants) suggest that social isolation impacts AD independent of loneliness [36], and loneliness has been suggested to interact with AD independent of social isolation [38]. Loneliness has not yet been reliably linked with brain volume changes, which suggests that the neurobiological associations between social isolation and AD are not shared by loneliness. Together, these factors suggest that loneliness may not contribute to AD as strongly as social isolation, which is why arrow ‘a' is marked thicker than arrow ‘c' in the model. The following sections of the review outline how social isolation and loneliness are distinct from one another.

### 2.2. AD-Relevant Neurobiological Processes in Social Isolation (Arrow ‘a')

#### 2.2.1. Initial Evidence in Humans

This section addresses that the underlying biological mechanisms present in social isolation (independent of loneliness) as they are relevant to AD. In the proposed model, this refers to arrow ‘a'.

Having a larger social network is correlated with reductions in brain tissue damage and neurofibrillary tau tangle load in autopsied brains from PLWAD [39]. Conversely, social isolation has been associated with various declining cognitive processes in the literature [40]. Socially isolated PLWAD present with decreased immediate and delayed recall abilities [41], increased levels of stress [22], and general cognitive decline (recently reviewed by [4] compared to nonisolated PLWAD), suggesting that social isolation contributes to AD pathoprogression. The neurobiological processes which contribute to these symptoms have been investigated using animal models.

#### 2.2.2. Neurological Processes in Social Isolation

Social isolation in rodents is directly linked many stages in AD pathogenesis including Aβ-amyloid plaque deposition [42, 43] and impaired tau phosphorylation [44]. Social isolation is also related through these processes to AD-like neuron cell death [45, 46] and reductions in new cell growth [47]. This process is similar to the AD pathogenic process involving neuroinflammatory pathways, which is observed more often in socially isolated than socially integrated rodents [46, 48]. When rodents are socially isolated, they present higher levels of inflammatory signals in the brain compared to nonisolated rodents [45, 46], which has been shown to increase the risk and severity of AD via the resident microglia (reviewed by [49]). The initial trigger to this AD-like pathogenic pathway is at least partly triggered by a chronic stress signal in the brain mediated by cortisol. Increased cortisol is observed when AD models are socially isolated and is linked with increased numbers of Aβ-amyloid plaques [50], which suggests that chronic social isolation-related stress is an important contributor to AD pathology.

#### 2.2.3. Social Isolation Is Stressful

Upregulation of stress pathways is an important element of the neurobiological processes in socially isolated individuals. Humans and other social animals including rodents become stressed when socially isolated for a long time [51], leading to chronic upregulation of stress pathways in the brain [52, 53]. It is important to note here that this stress response is more physiological than psychological. The level of cortisol upregulation is small but persistent when someone is socially isolated, which makes it unlikely that a person would experience a noticeable stress response. When a person is noticeably chronically stressed, the high level of cortisol upregulation induces a more profound inflammatory response which is associated with proximal and more damaging inflammatory disease [54].

Chronic upregulation of cortisol caused by social isolation, whether during old age or during development, is so closely related to AD pathology that it has been suggested to modulate clinical AD presentation in socially isolated humans and function as an AD biomarker [51]. Cortisol is thought to increase the levels of Aβ-amyloid plaque and associated biomarkers in the brain, as observed in rodents [55]. However, the precise mechanism here is not yet known [4]. Further understanding in this field would be useful not only for understanding the causal mechanism between social isolation and AD but also the way that stress contributes to neuroinflammation and potentially AD pathogenesis.

### 2.3. AD-Relevant Neurobiological Processes in Loneliness (Arrow ‘c')

Arrow ‘c' in the model represents processes by which loneliness contributes to AD pathology. Whilst social isolation is associated with brain changes in AD pathology, loneliness has not been directly linked, and associations between loneliness and AD are rare in the literature [8]. Studying biological effects of loneliness is difficult because of the reliance on animal models. Since socially isolated individuals often become lonely, the effects of loneliness on AD symptoms can be intertwined. Whilst it is easy to measure the effects of social isolation by removing peers from the environment, it is not possible to reliably measure loneliness independently of social isolation. Furthermore, variation in the way loneliness is measured makes interstudy comparison difficult. Loneliness in humans is typically measured either by a binary self-report item or using the three-item UCLA loneliness scale [56], which measure loneliness differently [57]. Loneliness measurement items are also employed at differing stages in assessment steps which are not readily measurable in large studies such as Shen et al. [36], which could produce serial order effects on the data [58]. It is difficult to quantify the effect of loneliness measurement discrepancies on the reliability of measuring loneliness. Nevertheless, high Aβ-amyloid plaque levels have been associated with a 7.5 times higher incidence of loneliness compared to baseline (using the UCLA scale) when controlling for other risk factors [59].

Furthermore, neuroimaging studies have linked loneliness (UCLA scale) to AD-like tau protein patterns in specific brain regions [44]. These results provide some good evidence that loneliness is associated with AD pathogenic processes. However, these patterns were not observed by Zhang et al. [5] using MRI-powered imaging. Whilst unable to show a relationship with AD pathology, Zhang and colleagues [5] observed evidence of brain atrophy consistent with general cognitive decline in lonely participants (measured by yes/no binary item), which is associated with later stages of AD. Another study using MRI was also able to associate loneliness (also measured by yes/no item) negatively with executive function and total brain volume and positively with white matter injury [60], which was suggested to mimic early AD pathology. Further results implicate loneliness in general brain degeneration processes [36], but these have not been experimentally linked to AD pathology. These links are useful to establish associations between loneliness and cognitive decline, but they cannot be used as evidence for a relationship with AD. Taken together, the existing evidence suggests that if loneliness has a direct neurobiological link, it may facilitate the beginning stages of AD. However, these results differ due to differences with loneliness metrics used, links to AD, and neurobiological measures, which hinder definitive conclusions. Much of the research provides neurobiological evidence for pathways which are active in AD but fail to link them to realised AD incidence. This is a clear opportunity for future research. Considering the current state of the literature, it is not possible to confidently identify a neurobiological pathway by which loneliness may directly affect AD.

### 2.4. Depression Might Explain Much of the Effect of Loneliness in the Context of AD (Depression and Loneliness Circles, Arrows ‘d', ‘e' and ‘f')

Current evidence does not clearly suggest a neurobiological association between loneliness and AD. This seems counterintuitive since loneliness can be linked to AD observationally [8, 41, 61]. The present review introduces depression as a possible explanatory modulating factor in the relationship.

In the model, depression and loneliness are linked with arrows ‘d' and ‘e'. This represents literature evidence that loneliness is often observed with depression, but depression can also lead to loneliness [62]. There is a very broad body of literature to establish these links [6, 17, 63–65], so here we focus on the central paradigm between loneliness, depression and AD (arrows d, e and f). As much as 75% of the relationship between loneliness and brain neurodegeneration is removed when depression is partialled out [36]. When specifically looking at the neurodegenerative processes in AD, depression explains up to 20% of risk when loneliness is included, but up to 50% when loneliness is partialled out [38]. Further, loneliness is not as directly related to Aβ-amyloid plaque formation or tau tangles as depression [38]. Therefore, based on the existing literature, depression is more closely related to AD than loneliness, and the effect of loneliness is at least partially moderated by depression. Consequently, in primary care, an indication of depression might be more important for AD risk than loneliness.

Loneliness has been associated with cognitive decline in older adults when depression is controlled for [66], but this relationship was not observed in the presence of AD. Further evidence like this may propose that a more complex model with at least one additional factor may better explain the relationship. In the case that future research provides convincing evidence that loneliness-driven cognitive decline is linked to AD, a role for loneliness in the intermediate to late stages of AD may be identified, and arrow ‘c' in the model would be thicker. For example, since loneliness contributes to cognitive decline, it may increase levels of chronic stress in the brain, which could contribute to the stress-driven process of AD pathology as discussed earlier. The existing literature in this area is very limited. The relationship between depression and social isolation is also outside the scope of this review, so there is no arrow between social isolation and depression in the model.

### 2.5. Depression and AD Risk (Arrow ‘f')

Evidence regarding depression's contribution to AD risk, which is represented by arrow ‘f' in the model, is reviewed in this section.

Depression is one of the primary contributors to and a prodrome of AD [67]. Whether depression is observed early in life or later, AD risk is increased and linked to neuron cell death in relevant brain regions [63, 68, 69]. Persistent depression likely increases AD risk significantly more than transient depression [69, 70], as does depression later in life compared to earlier presentation [67].

According to a review on the relationship between loneliness and depression in AD [71], depression has been implicated in brain degeneration and loss of function in many brain regions. This mechanism is driven by immune system dysregulation and has been proposed to be linked to immune cell activation in the brain by Wohleb [72]. Chronic stress may also play a role as a mechanistic driver in the proinflammatory pathway similar to what is observed in socially isolated brains [71]. Based on the observed patterns, this process could help to explain why depression is linked to AD risk [73], and how copresentation with loneliness could contribute to AD pathogenesis. Inflammatory depression is the most closely related type of depression, and antidepressant pharmaceuticals or practices can ameliorate and protect against damage in these pathways, depending on the type of antidepressant [67]. Clinical application here is clear; if depression is a stronger risk factor for AD than loneliness, and a clinician must choose to administer only one scale, depression measures are preferable as they may include loneliness effects. Further, interventions for depression may reduce loneliness-driven AD risk.

## 3. Discussion

The neurobiological evidence presented allows the drawing of two conclusions. First, a person experiencing social isolation is at higher risk for AD than a person experiencing loneliness. Second, when all other factors are stable, depression increases the AD risk in a person who is lonely.

The existing literature shows clearly that incidence of social isolation is often not directly related to loneliness. This suggests that although a person may be socially isolated, they may not experience feelings of loneliness, and a lonely person may not necessarily be socially isolated. As shown in the proposed model in Figure 1, it is useful to think of these conditions as distinct and sometimes copresenting. Clearly, a person who is both socially isolated and lonely likely has poorer health outcomes than a person who meets only one of these criteria, but because they are not directly linked, it is important to understand whether the lonely person who is socially active is more at risk for AD than the socially isolated person who is not lonely.

This paradigm has important implications for the dementia healthcare system, where much work is done to promote social interaction for people living with dementia and those at risk for dementia [74, 75]. Online interventions for older, socially isolated adults are impactful according to a scoping review from Rodrigues et al. [76]. According to the results, many socially isolated adults adopted coping behaviours including increases in messaging or calling loved ones. Tele-medicine including primary health services and psychological interventions provided much needed, but temporary, solutions. Importantly, older adults who struggle with contemporary technology were less likely to engage in these programs and were largely dependent on their cohabitants or nearby aged care home staff workers [76]. It is easy to understand that an older adult may remain socially isolated if they are not motivated toward pursuing social interaction and technological resources are out of reach.

Several components of the model may be altered if new findings come to light. In particular, the neurobiological underpinnings of loneliness are poorly understood, and whilst it is suggested that the literature currently does not support loneliness as a risk factor for AD, more research is required to elucidate the links between loneliness, depression and inflammatory- and stress-driven components of AD. In the model, this concerns arrows ‘c', ‘d' and ‘e'.

## 4. Clinical Implications

According to the existing evidence, a person who does not feel lonely but has very little social contact is likely at greater AD risk than a lonely individual who is socially integrated. In clinical practice and particularly from a psychological perspective, this might be counterintuitive. Typically, practitioners provide recommendations and therapeutics based on patient reports of distress. However, in this case, the undisclosed report may be more insidious. Socially isolated individuals who do not feel lonely, particularly in aged care homes, may be disproportionately at risk for AD. Practitioners should keep this in mind when appraising AD risk and severity and consider objective measures of social isolation over subjective measures like loneliness. Further, practitioners may consider measures of depression more appropriate for AD risk appraisal than measures of loneliness.

## 5. Conclusions

This review aimed to examine the evidence on the relationships between social isolation, loneliness, depression and the risk of developing AD. The secondary aim was to use the evidence to construct a new model on the contribution of social isolation and loneliness in the pathology of AD. The underlying neurobiological processes in social isolation and loneliness were reviewed, and the empirical evidence was organised to create a new model. Social isolation contributes to AD pathogenesis via neuroinflammation and stress pathways. Loneliness is linked to AD risk mainly through its strong association with depression. The proposed model provides a framework for future research in dementia risk factors and informs clinical appraisal of AD risk. More research is needed to fully understand this relationship. Since social isolation is linked more strongly with AD than loneliness, it may be more important to consider in AD risk. Further, older adults who are affected by social isolation are less likely to be identified if they are not also experiencing loneliness. Together, these contributors expose an under-recognised demographic of older adults who are socially isolated but not lonely, who may be at higher risk of AD.

## Data Availability

Data sharing is not applicable to this article as no datasets were generated or analysed during the current study.
